# Complete Genome Sequence of Staphylococcus aureus PS/BAC/169/17/W, Isolated from a Contaminated Platelet Concentrate in England

**DOI:** 10.1128/MRA.00841-21

**Published:** 2021-11-11

**Authors:** Carina Paredes, Sylvia Ighem Chi, Annika Flint, Kelly Weedmark, Carl McDonald, Jennifer Bearne, Sandra Ramirez-Arcos, Franco Pagotto

**Affiliations:** a Centre for Innovation, Canadian Blood Services, Ottawa, Canada; b Department of Biochemistry, Microbiology, and Immunology, University of Ottawa, Ottawa, Canada; c Bureau of Microbial Hazards, Health Products and Food Branch, Health Canada, Ottawa, Canada; d National Health Service Blood and Transplant, London, United Kingdom; University of Arizona

## Abstract

We report the genome sequence of Staphylococcus aureus PS/BAC/169/17/W, which was isolated in 2017 from a contaminated platelet concentrate at the National Health Service Blood and Transplant. Assessment of the genome sequence of this strain showed the presence of a 2,753,746-bp chromosome and a plasmid of 2,762 bp.

## ANNOUNCEMENT

Staphylococcus aureus, which is responsible for numerous infections (1, 2), is also a major contaminant of platelet concentrates (PCs) (3). Introduced into whole blood during blood collection, it can proliferate in manufactured PCs due to their storage conditions (4). S. aureus can escape detection during routine screening, causing false-negative transfusion reactions in recipients (3–6).

Here, the whole-genome sequence of S. aureus strain PS/BAC/169/17/W, which was isolated in 2017 from contaminated PCs by the National Health Service Blood and Transplant (NHSBT) (London, UK), is presented. S. aureus PS/BAC/169/17/W was isolated from contaminated PCs following standard procedures at the NHSBT and stored frozen at −80°C. For DNA isolation, S. aureus PS/BAC/169/17/W was streaked on blood agar plates. Single colonies were grown overnight at 35°C in 5 ml Trypticase soy broth with 0.6% yeast extract (7), collected by centrifugation, and resuspended in DNA/RNA Shield tubes (Cedarlane). DNA was extracted using the Zymo Quick-DNA high-molecular-weight (HMW) MagBead kit (Zymo Research Corp.) with lysozyme and RNAse A treatment according to the manufacturer’s manual. The same DNA extraction was used for both Nanopore and Illumina libraries.

Paired-end Illumina whole-genome shotgun (WGS) sequencing was performed using the Nextera XT DNA library preparation kit and a MiSeq instrument (v3 chemistry, 2 × 300-bp reads; Illumina, Inc.) according to the manufacturer’s instructions. Nanopore WGS sequencing libraries were constructed using the rapid barcoding sequencing kit (SQK-RBK004) and run using a FLO-MIN106 flow cell (R9.4) and a 1D MinION system (Oxford Nanopore Technologies) for 16 h according to the manufacturer’s protocol; signal processing, base calling, demultiplexing, and adapter trimming were performed using Guppy (Guppy GPU v 3.3.3+fa743ab).

Illumina reads (2,801,868 reads) were processed using fastp v0.20.0 (8) to remove adapter and barcode sequences, to correct mismatched bases in overlaps, and to filter low-quality reads, resulting into 2,644,066 filtered reads. Nanopore reads of <1 kb were removed using Filtlong v0.2.0 (https://github.com/rrwick/Filtlong), resulting in 246,318 filtered reads with an *N*_50_ value of 8,341 bp. Trycycler v0.3.3 (https://github.com/rrwick/Trycycler/wiki) (cluster, reconcile, partition, and consensus functions, with default circularization and rotation) was used for assemblies of the filtered Illumina and Nanopore reads. Briefly, Flye v2.8.1-b1676 (9), Minipolish and Miniasm v0.1.2 (10), wtdbg2 v2.5 (11), and Raven v1.2.2 (https://github.com/lbcb-sci/raven) were used to construct long-read assemblies, and a consensus genome was produced from the long-read assemblies using Trycycler cluster, reconcile, partition, and consensus functions. Error correction of the assembled genomes was performed using Medaka v1.1.3 (https://github.com/nanoporetech/medaka) followed by Pilon v1.23 (12) for Nanopore and Illumina reads, respectively. The average coverage for the assembled genome was 289× for the Illumina short-read data and 492× for the Nanopore long-read data. Genome annotation was performed using PGAP (release 2020-09-24.build4894; best-placed reference protein set) and GeneMarkS-2+ (https://github.com/ncbi/pgap) and assessed using QUAST v5.0.2 (https://github.com/ablab/quast). All versions, citations, and nondefault settings are included.

The closed PS/BAC/169/17/W genome has an average GC content of 32.84% and includes a closed, circular 2,753,746-bp chromosome and a single, circular, 2,762-bp plasmid, containing a total of 2,507 genes, 133 pseudogenes, 0 CRISPRs, 19 rRNAs, 58 tRNAs, and 4 noncoding RNAs. PS/BAC/169/17/W was assigned to sequence type 582 (ST582) and clonal complex 15 (CC15) using the PubMLST database (13).

### Data availability.

This genome is available in GenBank and the Sequence Read Archive (SRA) under the accession numbers indicated in Table 1.

**TABLE 1:** Provenance and NCBI accession numbers for the PS/BAC/169/17/W isolate

Parameter	Details
Isolate	PS/BAC/169/17/W
BioProject accession no.	PRJNA703966
GenBank accession no.	
Chromosome	CP071100
Plasmid	CP071101
SRA accession no.	
Illumina reads	SRR13745243
Nanopore reads	SRR13745249
Country (region)	United Kingdom (England)
Year	2017
